# RETRACTED: Mansoor et al. Effect of Currently Available Nanoparticle Synthesis Routes on Their Biocompatibility with Fibroblast Cell Lines. *Molecules* 2022, *27*, 6972

**DOI:** 10.3390/molecules31214002

**Published:** 2026-11-06

**Authors:** Afsheen Mansoor, Zohaib Khurshid, Emaan Mansoor, Muhammad Talal Khan, Jithendra Ratnayake, Asif Jamal

**Affiliations:** 1 Department of Dental Material Sciences, School of Dentistry, Shaheed Zulfiqar Ali Bhutto Medical University, Islamabad 45320, Pakistan; 2 Department of Prosthodontics and Dental Implantology, College of Dentistry, King Faisal University, Al-Ahsa 31982, Saudi Arabia; 3 Islamic International Dental College, Riphah International University, Islamabad 6000, Pakistan; 4 Department of Dental Biomaterials, Bakhtawar Amin Medical and Dental Collage, Multan 60650, Pakistan; 5 Department of Oral Sciences, Faculty of Dentistry, University of Otago, Dunedin 9016, New Zealand; 6 Department of Microbiology, Quaid-i-Azam University, Islamabad 45320, Pakistan

The journal retracts the article “Effect of Currently Available Nanoparticle Synthesis Routes on Their Biocompatibility with Fibroblast Cell Lines” [1] cited above.

Following publication of a correction to this article [1], additional concerns were brought to the attention of the Editorial Office regarding the reliability of the corrected Figure 15, as well as further potential issues relating to images, graphs, data, methodology, and citations.

In accordance with standard journal procedures, the Editorial Office and Editorial Board conducted an investigation, which included a reassessment of Figure 15. The investigation identified indications of inappropriate editing and duplication between Figure 2 and an earlier publication [2], published by a similar authorship group, presenting different experimental conditions. In addition, the Editorial Board confirmed concerns relating to the validity of Figure 15, the accuracy of the data represented in Table 1, and Figures 5, 6, 7, 10, 11, 12, 13 and 14, and the appropriateness of methodology, as well as the inclusion of a significant number of inappropriate citations. Consequently, the Editorial Board has lost confidence in the reliability of the findings and decided to retract this publication [1], as per MDPI’s retraction policy.

This retraction was approved by the Editor-in-Chief of the journal *Molecules*.

The authors have been informed of this retraction and have indicated their disagreement with this decision.
